# Effects of the solubility of yeast cell wall preparations on their potential prebiotic properties in dogs

**DOI:** 10.1371/journal.pone.0225659

**Published:** 2019-11-25

**Authors:** Stephanie de Souza Theodoro, Thaila Cristina Putarov, Caroline Tiemi, Lara Mantovani Volpe, Carlos Alberto Ferreira de Oliveira, Maria Beatriz de Abreu Glória, Aulus Cavalieri Carciofi

**Affiliations:** 1 Veterinary Medicine and Surgery Department, College of Agrarian and Veterinarian Sciences (FCAV), São Paulo State University–UNESP, Jaboticabal, São Paulo, Brazil; 2 Biorigin Brasil, Lençois Paulistas, São Paulo, Brazil; 3 Pharmacy Faculty, Minas Gerais Federal University (UFMG), Belo Horizonte, Minas Gerais, Brazil; USDA-Agricultural Research Service, UNITED STATES

## Abstract

Derivatives of yeast cell wall (YCW) have been studied for their potential prebiotic effects. Recently, new purified and soluble preparations have been developed in an attempt to increase their biological actions. Two YCW preparations, one conventional and another with higher solubility of the mannan oligosaccharide fraction, were evaluated on dogs. One food formulation was used, divided into the following treatments: CON–control, without yeast cell wall addition; YCW–addition of 0.3% of a conventional yeas cell wall extract; YCWs–addition of 0.3% of a yeast cell wall extract with high mannan oligosaccharide solubility. Twenty-four beagle dogs were used, eight per food, distributed on a block design. Blocks lasted 32 days, and TNF-a, IL-6, IL-10, *ex vivo* production of hydrogen peroxide and nitric oxide by peripheral neutrophils and monocytes, phagocytic index, and fecal IgA were evaluated at the beginning and end of each period. Additionally, nutrient digestibility, feces production and quality, and fermentation products were quantified. The results were evaluated by analysis of variance and compared using the Tukey test (P<0.05), using the basal immunological parameters as a covariate. The inclusion of YCWs reduced fat digestibility (P<0.05), increased the concentration of butyrate and putrescine, and reduced lactate in feces (P<0.05), showing that mannan oligosaccharide solubilization resulted in higher fermentation of this compound and altered the metabolism of the gut microbiota. Lower IL-6 on serum was verified for dogs fed the YCWs diet (P<0.05), suggesting a reduction in the inflammatory activity of dogs. Higher phagocytic index was verified for peripheral monocytes after the intake of the YCW food, suggesting better innate immunity. In conclusion, the solubilization of the mannooligosaccharide fraction alters its interaction with gut microbiota and biological actions in animals, although both yeast cell wall preparations exhibited prebiotic effects on dogs.

## Introduction

The health of the gut is dependent on a dynamic interrelationship between the gut microbiota and gut nutrition [1,2], reflecting directly on the immunological status and general health of dogs [3,4]. It is postulated that the intestinal microbiota performs at least three main functions: protection, nutrition and metabolic control [5]. The microbiota acts as a barrier with important protective effect against pathogens; performs the fermentation of dietary nondigestible residues and endogenous substances, allowing the production of important nutrients for gut mucosa such as short-chain fatty acids; controls the proliferation and differentiation of intestinal epithelial cells; and contributes to immune system development and homeostasis [5].

Because intestinal microbes subsist on products resulting from the interaction between the host and its diet, food composition is one of the most important factors for gut microbiota maintenance, structure and function [1,6,7]. In this regard, yeast cell wall (YCW) may be an important energy source for intestinal microorganisms [8] and has been studied as a prebiotic candidate for dogs [9,10]. Mainly composed of carbohydrates and proteins, their main chemical constituents are mannose, glucose and N-acetylglucosamine (chitin) [11,12]. The YCW apparently meets the three essential criteria of a prebiotic [13], it is resistant to gastric acidity and hydrolysis by mammalian enzymes and to gastrointestinal absorption, is fermented by intestinal microbiota, and selectively stimulates the growth and/or activity of intestinal bacteria associated with health and wellbeing [1,14,15].

Among the possible mechanisms implicated for host health, prebiotics such as the YCW may promote short chain fatty acid (SCFA) production, colon pH regulation, and competition against pathogens for cell mucosa receptors [16]. Experimental data on animal studies have shown that the gut-associated lymphoid tissue (GALT) may be the primary target of the immunomodulatory effect of prebiotics [17,18], and the enterocytes are key intermediates that transmit signals from the intestinal lumen to the GALT [18]. Increase in serum lymphocyte concentration and decline in plasma neutrophils was reported in dogs fed YCW, indicative of an improvement in immunological status [19]. However, most publications on dogs only evaluated digestibility and fermentation products, and few evaluated the effects of the YCW on immunity. The SCFA generated after microbial fermentation of the YCW components may also modulate inflammation, since butyric acid may inhibit the production of the proinflammatory cytokines IL-2 and IFN-γ, and acetic and propionic acids may increase the production of the immunoregulatory cytokine IL-10 [20,21].

In recent years, specific strains of *Saccharomyces cerevisiae* and special techniques to separate and purify specific components of the cell wall structure have been developed. More purified than conventional YCW derivates, which consist of simple dried cell walls after the cellular content removal, these preparations have higher concentrations of soluble mannan oligosaccharides, smaller particle size and higher solubility in water, which are characteristics that may influence YCW exposure to gut microbiota and the host mucosa, potentially inducing different biological responses [10,22]. Based on these developments, the present study evaluated the effects of the incorporation in extruded diets of two preparations of *Saccharomyces cerevisiae* cell wall, differing in solubility in water of mannan oligosaccharides, on nutrient digestibility, microbial fermentation products in feces, and certain immunological parameters of adult dogs.

## Experimental methods

The study was conducted in the Laboratory of Research in Nutrition and Nutritional Diseases of Dogs and Cats, College of Agrarian and Veterinarian Sciences, Sao Paulo State University (UNESP), Jaboticabal, SP, Brazil. All procedures with animals followed the ethical principles adopted by the Brazilian College of Animal Experimentation and were previously approved by the Ethics Committee on the Use of Animals (protocol number: 011937/17).

### Test products

Two yeast derivates were used, obtained by the industrial purification of *Saccharomyces cerevisiae* cell wall (Biorigin, Lençóis Paulista, Sao Paulo, Brazil). After industrial purification, the yeasts culture was submitted to autolysis where intracellular enzymes are activated by appropriate processing conditions resulting in a partial degradation of the cell wall structures, followed by centrifugation and separation of the yeast extract from the yeast cell wall [23]. By this processing the standard purified Yeast Cell Wall (YCW) product was obtained with a water solubility index of approximately 20% (Table 1). Further, the purified yeast cell wall was submitted to a processing of chemical hydrolysis by acids [24,25], in order to partially solubilize the mannan-protein outer layer to obtain the soluble Yeast Cell Wall (YCWs) product, which presented 40% of water solubility index. Mainly mannan oligosaccharides were solubilized during the preparation of the extract, resulting in 2.1% soluble mannan oligosaccharides on the YCW and 22.2% soluble mannan oligosaccharides on the YCWs. The water solubility index was determined as previously described [26].

**Table 1:** Characteristics of the yeast cell wall derivates used on the study.

Item	Yeast Cell Wall preparations ^2^	
	YCW	YCWs
Moisture (%)	6.9	4.5
Protein (%)	31.4	25.4
Ash (%)	7.7	5.4
pH	4.9	3.6
Total mannan oligosaccharides (%)	17.1	23.9
Soluble mannan oligosaccharides^1^ (%)	2.1	22.2
Glucans (%)	25.1	24.9
Water solubility index^1^	19.6	42.6

^**1**^ Determined on Biorigin Laboratory (Lençois Paulistas, Brazil) [23].

^2^ YCW = standard yeast cell wall extract; YCWs = yeast cell wall extract with 20% soluble mannan oligosaccharides.

### Animals

Twenty-four adult Beagle dogs, males and females, with 3.5±0.91 years of age and weighing 11.95±1.12 kg were used. All animals belong to the kennel of the Laboratório de Pesquisa em Nutrição e Doenças Nutricionais de Cães e Gatos, FCAV/UNESP, Jaboticabal, Brazil. The mean body condition score of the dogs was 6.0±1.2, on a scale from 1 to 9 [27]. Prior to the study, dogs were submitted to physical, hematological, and serum biochemical evaluations by a veterinarian, and all were considered healthy.

### Experimental design

The study included three experimental diets and was conducted in a randomized block design with two blocks of 12 dogs each and four dogs per diet in each block, totaling eight animals (repetitions) per diet (treatment). The blocking factor was time, due to available structure for research. Each block lasted 32 days and included testing the following: phagocytic activity of peripheral monocyte and neutrophils were evaluated on days 0, 15 and 30; cytokines in peripheral blood and the *in vitro* production of hydrogen peroxide and nitric oxide in cell culture were evaluated on days 0 and 30; IgA content in feces was evaluated prior to study (feces collected from days -2 to 0) and after 30 days of diet intake (days 30 to 32); total feces collection for digestibility measurement was performed from days 16 to 20; fresh feces collection to analyze fermentation products, pH and biogenic amines was conducted on days 23 to 25.

The amount of food offered was initially calculated considering the food metabolizable energy content, estimated by its chemical composition, and the individual energy requirement of laboratory dogs [28]. The daily amount was provided once a day (at 10 am). Offered and refused food was weighed, and the intake was recorded. Dogs were then weighed weekly, and the amount of food provided adjusted such that animals maintained a constant body weight throughout the study. Water was provided *ad libitum*. During the study dogs were housed in kennels measuring 1.5 m x 3.5 m with a solarium, and released daily in a collective playground for exercise and socialization.

### Experimental diets

A single formulation based on corn grain, poultry byproduct meal, poultry fat and sugarcane fiber was used (Table 2), balanced for adult dogs according to the nutritional recommendations of the European Pet Food Industry Federation [29]. Sugarcane fiber was used due to its low fermentation [30], reducing interference with formation of fermentation products. The experimental diets were obtained by the addition of the different yeast cell wall extracts, added in replacement of corn (on an as-fed basis): CON—control diet, without inclusion of yeast cell wall extract; YCW—inclusion of 0.30% of YCW; YCWs—inclusion of 0.30% of YCWs.

**Table 2:** Ingredient and chemical composition of the food used on the study.

Ingredients	%
Corn grain	51.6
Poultry by-product meal	32.2
Poultry fat	9.2
Liquid palatant ^1^	3.0
Sugarcane fiber ^2^	2.0
Vitamin-mineral premix ^3^	0.5
Salt	0.5
Potassium chloride	0.5
Choline chloride	0.3
Mold inhibitor ^4^	0.1
Antioxidant ^5^	0.04
L-lysine	0.03
Analyzed chemical composition	%, as fed basis
Dry matter	93.0
Crude protein	27.4
Ash	9.0
Acid-hydrolyzed fat	17.4
Crude fiber	2.6
Calcium	1.9
Phosporus	1.3

^1^ D’TECH 10L, Palatabilizante Líquido, SPF do Brasil Indústria e Comércio Ltda., Descalvado, Brazil.

^2^ Vit2be Fiber, Dilumix Industrial Ltda., Leme, Brazil.

^3^ Rovimix, DSM Produtos Nutricionais Brasil S.A., Jaguaré, Brazil. Added per kg of food: Vitamin A, 18,750 IU; Vitamin D3, 1,500 IU; Vitamin E, 125 IU; Vitamin K3, 1,5 mg; Vitamin B1, 5 mg; Vitamin B2, 16.25 mg; Pantothenic Acid, 37.5 mg; Vitamin B6, 7.5 mg; Vitamin B12, 45 mcg; Vitamin C, 0,125 g; Nicotinic Acid, 0.0625; Folic Acid, 0.75 mg; Biotin, 0.315 mg; Iron, 0.1 g; Copper, 9.25 mg; Manganese, 6.25 mg; Zinc, 0.15 g; Iodine, 1.875 mg; Selenium, 0.135 mg.

^4^ Mold-Zap Citrus, Alltech do Brasil Agroindustrial Ltda., Araucária, Brazil.

^5^ Banox, Alltech do Brasil Agroindustrial Ltda., Araucária, Brazil.

Dietary formulations were processed at the Extrusion Laboratory of the College of Agrarian and Veterinarian Sciences, Sao Paulo State University (UNESP), Jaboticabal, SP, Brazil. A single lot of raw materials was used for the three experimental diets. Ingredients were weighed and mixed before being ground in a hammer mill (Tigre, Moinhos Tigre, São Paulo, SP), fitted with a 0.8 mm sieve screen size, and extruded in a single-screw extruder (Model Mex-250, Manzoni Industria Ltda, Campinas, SP), with an average extrusion capacity of 250 kg/h. The temperature of the extruder preconditioner was kept higher than 85°C by direct steam injection. After extrusion, the kibbles were dried in a forced air dryer at 105°C for approximately 20 min and coated with poultry fat and liquid palatant.

### Digestibility protocol, feces production and characteristics

This evaluation followed recommendations and procedures previously described [29]. Dogs were individually housed for 5 days in stainless steel metabolic cages, and each contained an apparatus to collect feces and urine separately. Food consumption was recorded daily, measuring the offered and refused amounts. Feces were quantitative collected at least twice a day for 120 h, weighed, and stored frozen at -15°C until analysis. After the end of the collection period, feces were thawed to room temperature and homogenized, compounding a single sample per dog, and then they were dried in a forced-air oven (320-SE, FANEM, São Paulo, Brazil) at 55°C for 72 hours. Predried feces and diets were ground in a knife type mill (MOD 340, ART LAB, São Paulo, Brazil) with a 1 mm sieve for laboratory analysis.

The gross energy (GE) content of diets and fecal samples was determined using a bomb calorimeter (IKA C2000 Basic, IKA-Werke GmbH & Co. KG, Staufen, Germany). Dry matter (DM) was determined by oven-drying the sample (method 934.01), ash was measured by muffle furnace incineration (method 942.05), crude protein was estimated using a LECO nitrogen/protein determination (FP-528, LECO Corporation, Saint Joseph, USA; method 990.03), total fat was assessed using the acid-hydrolyzed fat assay (method 954.02), and organic matter (OM) was calculated as DM minus ash. All samples were analyzed in duplicate, and the analyses were repeated when the variation between replicates was greater than 5%.

Fecal score was determined using the following system [31]: 0 = watery liquid that can be poured; 1 = soft, unformed; 2 = soft, malformed stool that assumes the shape of its container; 3 = soft, formed, and moist stool that retains its shape; 4 = well-formed and consistent stool that does not adhere to the floor; and 5 = hard, dry pellets, which are small and hard masses.

### Fecal pH and fermentation products

For this evaluation, from days 23 to 25 for each block fecal samples were collected immediately after elimination for three consecutive days. Fecal pH was determined for 2 g of fresh feces mixed with 6 mL of ultrapure water, using a pH meter (model DM20; Digicrom Analitica LTDA, São Paulo, Brazil). Approximately 10 g of fresh feces was homogenized and mixed with 30 mL of a 16% (vol/vol) formic acid solution and precipitated to determine the volatile fatty acids (VFA). Next, the mixture was centrifuged (5810R; Eppendorf, Hamburg, Germany) 3 times at 4,500 x g for 15 min at 4°C. The supernatant was retained, and the pellet was discarded. The short-chain fatty acids (SCFA) and branched-chain fatty acids (BCFA) of the supernatant were determined by gas chromatography (model 9001; Finnigan Corporation, San Jose, CA) as previously described [32]. Lactic acid was measured by mixing 3 g of feces with 9 mL of distilled water. This mixture was centrifuged 3 times at 4,500 x g at 4°C for 15 min. The supernatant was obtained, and the pellet was discarded. The analysis of lactic acid was performed by spectrophotometry (Spectrophotometer Quick-Lab; Drake Eletronica e Comércio, São José do Rio Preto, São Paulo, Brazil) [33]; samples were quantified by comparing them with a standard curve for lactic acid. The concentration of ammonia was assessed in the same extracts prepared for the VFA. The extracts were thawed at room temperature, and 2 mL of each extract was diluted into 13 mL of distilled water and submitted to distillation in a nitrogen system (Tecnal TE-036/1; Tecnal Equipamentos Científicos, Piracicaba, São Paulo, Brazil).

To determine concentrations of biogenic amines in feces, five grams of fresh feces was homogenized and added to 7 mL of a 5% trichloroacetic acid solution and then mixed for 3 min by vortex and centrifuged at 10,000 x g for 20 min at 4°C (5810R; Eppendorf, Hamburg, Germany) [34]. The supernatant was filtered with qualitative filter paper, and the residue was extracted twice using 7 and 6 mL of a 5% trichloroacetic acid solution, separately. Then, the supernatants were filtered and pooled. The final volume obtained was recorded and frozen. Biogenic amine concentrations were determined in the supernatant by HPLC (HPLC model LC-10AD; Shimadzu Corporation, Kyoto, Japan).

### Fecal Ig A

Fresh feces (immediately after elimination) was collected for three consecutive days before and 30 days after the intake of the experimental diets. For each period, fecal samples were pooled by dog, and fecal IgA was extracted using a saline solution [35]. Approximately 1 gram of feces was weighed and diluted in 10 mL of extraction buffer composed of 0.01 M phosphate-buffered saline (PBS) (pH 7.4), 0.5% Tween (Sigma-Aldrich, St Louis, MO, USA), and 0.05% sodium azide. After homogenization, the fecal suspensions were centrifuged at 1,500 x g for 20 min at 5°C. Then, 1 mL of the supernatant was transferred to a sterile microtube containing 20 μL of a protease-inhibitor cocktail (Sigma-Aldrich, St Louis, MO, USA). To remove the residues, samples were centrifuged at 15,000 x g for 15 min at 5°C, and the supernatants were kept in microtubes at -20°C until analysis.

The quantification of IgA was performed by an ELISA kit for canine IgA determination (Bethyl Laboratories, Montgomery, TX, USA). Optical density (OD) was read at 450 nm with a Microplate Reader (MRX TC Plus, Dynex Technology, Chantilly, Virginia, EUA). To calculate the IgA concentration, the OD of the samples was compared to the OD of a standard with a known concentration of IgA. The standard canine IgA sample was provided in the kit, and seven dilutions of the standard were made in order to develop a regression curve between OD and IgA amount. Samples were analyzed in duplicate, and the analysis was repeated when the variation between replicates was greater than 10%.

### TNF-α, IL-6 and IL-10 on blood serum

For analyses of tumor necrosis factor alpha (TNF-α) and interleukins 6 (IL-6) and 10 (IL-10), on days zero and 30 blood samples (3 mL) were collected via jugular puncture and placed in tubes without anticoagulant. Afterwards, the samples were centrifuged at 3,500 x g for 10 min (5810R; Eppendorf, Hamburg, Germany), and the serum was stored frozen at -80°C until analysis. The dosage was estimated using a Luminex kit specific to dogs, according to the manufacturer's recommendations (MILLIPLEX MAP ELISA Canine Cytokine / Chemokine Magnetic Bead Panel—Immunology Multiplex Assay—Merck Millipore, St Charles-Missouri-USA).

### Phagocytic activity

Phagocytic activity was measured on days 0, 15 and 30 using a commercial kit (pHrodo *E*. *coli* BioParticles, Molecular Probes Inc., Oregon, USA). Blood samples were collected by jugular puncture and placed in heparinized tubes. Then, 100 μL of each sample was incubated with 20 μL of pHrodo *E*. *coli* BioParticles, a reagent provided by the commercial kit. For each blood sample two tubes were prepared with the bioparticles; one was placed on ice, and the other kept in a water bath at 37 ºC for 15 min. Next, the incubated samples were lysed, followed by centrifugation and washing using the proper reagents as recommended by the manufacturer. Two negative control samples were run together on each collection day, both tubes with no bioparticles, but one placed on ice and the other kept at 37 ºC. Samples were analyzed using a flow cytometer (FACSCanto, Becton Dickinson Immunocytometry System, Mountain View, CA, USA), and the results were expressed as the percentage of fluorescence signal inside the desired population of neutrophils and monocytes. The target cell population was gated according to its volume and complexity [36].

### Determination of hydrogen peroxide (H_2_O_2_) and nitric oxide (NO) production

Blood samples (6 mL) were collected with heparin via jugular puncture and added to 4.5 mL of Histopaque 1119 and 3 mL of Histopaque 1077 (Sigma Aldrich, St Louis, MO, EUA) in 15-mL conical centrifuge tubes. Tubes were centrifuged at 700 x g for 30 min at room temperature. After centrifugation, two distinct opaque layers separated, the mononuclear and granulocyte cells. Each layer was collected separately, transferred to a 50-mL conical centrifuge tube and washed at least twice with isotonic phosphate buffered saline by centrifugation at 360 x g for 10 min at room temperature. Erythrocyte lysis was conducted when necessary using 2 mL of ACK (Ammonium-Chloride-Potassium) solution (0.15 M ammonium chloride; 10 mM potassium bicarbonate; 0.1 mM EDTA) for a maximum of 2 min.

Cells were suspended in complete medium (RPMI 1640, Merck KGaA, Darmstadt, German), added to 40 mg/mL gentamicin and 10% fetal bovine serum, and the concentration was adjusted to 2 x 10^5^ neutrophils or monocytes/mL. Then, suspensions were placed in 96-well flat plates (100 μL/well). Mononuclear cells were kept at 37°C in a humidified 5% CO_2_ incubator (Thermo Fisher Scientific, Waltham, MA, USA) for one hour for monocytes to adhere to the well surfaces, then supernatants were carefully discarded with a pipette and complete medium was added to each well. For monocytes, one plate was incubated for H_2_O_2_ production analysis and one for NO production analysis. For neutrophils, the supernatant from H_2_O_2_ production was used to conduct the NO analysis.

For H_2_O_2_ production, a total of 24 wells received 100 μL of sample; 12 of them were maintained as suspensions of nonstimulated cells, and the others 12 wells were stimulated with LPS (1 μg/well—*E*. *coli Lipopolysaccharide*, Sigma Aldrich, St. Louis, USA). Plates were maintained at 37°C in a humidified 5% CO_2_ incubator for 36 hours. The H_2_O_2_ production was measured as previously described [37,38]. Buffer solution (100 μL/well) consisting of 7.8 mL distilled water (dH_2_O), 0.8 mL of solution A (800 mL dH_2_O, 80 g NaCl, 2 g KCl, 2 g KH_2_PO_4_, 11.5 g Na_2_HPO_4_), 0.1 mL of solution B (100 mL dH_2_O, 1 g CaCl_2_), 0.1 mL of solution C (100 mL dH_2_O, 1 g MgCl_2_)_,_ 0.1 mL of phenol red (100 mL dH_2_O, 1 g phenol red), 0.1 mL of peroxidase (10 mg horseradish peroxidase, 2 mL PBS) and 1 mL of glucose (100 mL dH_2_O, 1 g glucose), was added to each well. Phorbol myristate acetate (PMA, 10 μL/well) was added to half of the nonstimulated wells and to half of LPS-stimulated wells and kept at 37°C in a humidified 5% CO_2_ incubator for one hour. Consequently, there were six replications for each cell condition: six wells for nonstimulated cells, six wells for nonstimulated + PMA, six wells for LPS-stimulated cells, and six wells for LPS-stimulated cells + PMA. After one-hour incubation, the reaction was stopped with 10 μL of 1 N NaOH. The absorbance was read in a microplate reader (iMarkMicroplate Absorbance Reader 168–1135, Bio-Rad, Hercules, California, USA) at 595 nm. The results were expressed in μM amounts of H_2_O_2_/2x10^5^ cells. A hydrogen peroxide standard curve was constructed for each plate with a range of 0.25 to 16.00 nM of H_2_O_2_.

The NO production was assessed by the colorimetric method of the GRIESS reaction [39]. The analyses were conducted in six repetitions for nonstimulated cells and six repetitions for LPS-stimulated cells (1 μg/well *E*. *coli Lipopolysaccharide*, Sigma Aldrich, St. Louis, USA), totaling 12 wells per sample. One-hundred μL of GRIESS reagents diluted 1:1 (n-(1-naftil)-etil-enediamin diluted 0.1% in dH_2_O, 1% sulfonamide diluted in 5% H_2_PO_4_, Sigma Aldrich, St Louis, MO, USA) were added to the supernatant. The absorbance was read in a microplate reader (iMarkMicroplate Absorbance Reader 168–1135, Bio-Rad, Hercules, California, USA) at 540 nm. The results were expressed as μM amounts of NO/2x10^5^ cells. An NO standard curve was constructed for each plate with a range of 0.78 to 100 μM of NO.

### Statistical analysis

All variables were previously tested for normality or errors using the Cramer-von Misses test and for homoscedasticity using the Levene test. When necessary, logarithmic transformation (log x + 1) or lambda transformation was applied. For the immunological parameters, data were submitted to analysis of variance considering the effects of block, animal and diet. Differences among groups was detected at baseline, and for this reason the time 0 (baseline) was used as a covariate. When results of the F-test were significant, multiple comparisons of the means were made using Tukey’s test. Data obtained for nutrient digestibility, fecal parameters and fermentation products were submitted to analysis of variance and, when significant, compared by Tukey's test (P<0.05). Values of P<0.05 were considered significant, and P<0.10 as a trend. The analysis was conducted using the computer program R (version 3.3.3).

## Results

Dogs showed proper food intake and maintained a constant body weight throughout the experimental period, with no episodes of food rejection, vomiting, or diarrhea. The food intake did not differ among diets (P>0.05). For the digestibility evaluation, DM intake was similar, resulting in similar nutrient intake by the animals (Table 3). The total tract apparent digestibility of nutrients was similar, except that fat digestibility was lower for dogs fed the YCWs food (P<0.05) than for CON. Feces production, DM, score and pH were also similar among treatments (P>0.05), as shown in (Table 4).

**Table 3:** Body weight (kg), nutrient intake (g/dog/day) and coefficients of total tract apparent digestibility (%) of nutrients of diets for dogs with the additions of different yeast cell wall preparations (mean and standard error of the mean).

Item	Diets^1^			*p-value*
	CON	YCW	YCWs	
Body weight (kg)	11.6±0.1	11.7±0.1	11.6±0.1	0.677
Nutrient intake (g/dog/day)				
Dry matter	160.4±11.3	170.6±12.9	163.2±9.8	0.317
Coefficient of total tract apparent digestibility (%)				
Dry matter	80.4±1.2	79.7±1.7	78.5±3.9	0.437
Organic matter	85.5±0.9	85.0±1.3	84.0±3.0	0.398
Crude protein	85.1±2.1	84.0±2.3	83.1±3.0	0.274
Fat	94.3±0.6^a^	92.4±1.6^ab^	91.6±2.1^b^	0.014
Gross energy	85.3±1.0	84.8±1.7	84.8±2.8	0.847

^1^ CON = control, without yeast cell wall addition; YCW = 0.3% of a standard yeast cell wall extract; YCWs = 0.3% of a yeast cell wall extract with 20% soluble mannan oligosaccharides.

^a, b^ = means in a row without a common superscript letter differ (P<0.05).

**Table 4:** Feces production and characteristics of dogs fed diets with the addition of different yeast cell wall preparations (mean and standard error of the mean).

Feces	Diets^1^			*p-value*
	CON	YCW	YCWs	
g/dog/day (as is)	75.6±8.6	81.5±19.3	86.0±15.3	0.697
g/dog/day (dry matter basis)	33.8±2.2	37.3±5.1	37.9±7.9	0.434
Dry matter (%)	45.1±3.8	46.1±3.1	44.5±6.2	0.782
Score	3.6±0.3	3.8±0.2	3.7±0.3	0.378
pH	6.8±0.2	6.9±0.1	6.8±0.1	0.172

^1^ CON = control, without yeast cell wall addition; YCW = 0.3% of a standard yeast cell wall extract; YCWs = 0.3% of a yeast cell wall extract with 20% soluble mannan oligosaccharides.

For fermentation products, higher concentrations of butyrate (approximately 25% more) and lower lactate (approximately 64% less) were verified in the feces of dogs fed the YCWs than in the other two foods (P<0.05), and there were no other detectable differences (Table 5).

**Table 5:** Volatile fatty acids (mMol/g of dry matter), ammonia and lactic acid concentration (mMol/kg of dry matter) on the feces of dogs fed diets with the addition of different yeast cell wall preparations (mean and standard error of the mean).

Item	Diets^1^			*p-value*
	CON	YCW	YCWs	
Acetic acid	230.3±50.0	218.4±30.3	230.0±55.2	0.851
Propionic acid*	111.4±22.6	101.9±18.9	108.1±27.7	0.739
Butyric acid	46.8^b^±4.9	43.7^b^±6.4	55.6^a^±7.1	0.004
Isobutyric acid	10.1±1.4	9.6±1.0	10.2±2.4	0.808
Isovaleric acid	14.3±2.1	12.7±1.8	14.6±2.7	0.301
Valeric acid	2.7±1.2	3.5±1.5	3.4±1.1	0.138
Total VFA^2^*	417.9±79.0	389.8±51.0	421.9±87.5	0.775
Total SCFA^3^*	390.8±76.3	364.0±49.0	393.7±86.7	0.801
Total BCFA^4^	27.1±3.8	25.8±3.9	28.2±4.6	0.595
Ammonia	194.5±24.8	174.4±20.4	196.1±32.0	0.171
Lactic acid*	2.7^a^±0.7	2.9^a^±0.8	1.7^b^±0.3	0.010

^1^ CON = control, without yeast cell wall addition; YCW = 0.3% of a standard yeast cell wall extract; YCWs = 0.3% of a yeast cell wall extract with 20% soluble mannan oligosaccharides.

^2^: VFA = volatile fatty acids.

^3^: SCFA = short-chain fatty acids.

^4^: BCFA = branched-chain fatty acids.

* Values transformed to Log x + 1 or γ for statistical analysis.

^a, b^ = means in a row without a common superscript letter differ (P<0.05).

Only the biogenic amines putrescine, cadaverine, spermidine and phenylethylamine were detected in significant amounts in the dog feces (Table 6). Among them, putrescine was approximately 70% higher for dogs fed the YCWs than for those fed CON (P<0.05). Additionally, the feces of dogs fed the YCWs diet tentend to present higher spermidine concentration than CON (P = 0.096). The fecal concentration of IgA was similar between dogs fed the experimental foods, as presented in (Table 7).

**Table 6:** Biogenic amines concentration (mg/100 g of dry matter) on the feces of dogs fed diets with the addition of different yeast cell wall preparations (mean and standard error of the mean).

Item	Diets^1^			*p-value*
	CON	YCW	YCWs	
Putrescine*	9.8±4.9^b^	12.3±6.3^ab^	22.3±12.9^a^	0.035
Cadaverine*	4.2±3.9	3.3±1.5	5.2±3.8	0.469
Spermidine	2.3±1.2	3.0±1.4	3.4±1.6	0.096
Phenylethylaminev	0.9±0.8	1.1±0.8	1.1±0.8	0.286

^1^ CON = control, without yeast cell wall addition; YCW = 0.3% of a standard yeast cell wall extract; YCWs = 0.3% of a yeast cell wall extract with 20% soluble mannan oligosaccharides.

* Values transformed to Log x + 1 or γ for statistical analysis.

^a, b^ = means in a row without a common superscript letter differ (P<0.05).

**Table 7:** Immunoglobulin A concentration (mg/g of dry matter) on the feces of dogs fed diets with the addition of different yeast cell wall preparations (mean and standard error of the mean).

Item	Diets^1^			*p-value*
	CON	YCW	YCWs	
IgA*	3.1±1.4	3.7±1.3	2.5±1.5	0.945

^1^ CON = control, without yeast cell wall addition; YCW = 0.3% of a standard yeast cell wall extract; YCWs = 0.3% of a yeast cell wall extract with 20% soluble mannan oligosaccharides.

* Values transformed to Log x + 1 or γ for statistical analysis.

Among the evaluated cytokines (Table 8), dogs fed the YCWs diet exhibited lower IL-6 serum concentration than did animals fed the CON diet (P<0.05), but similar values in comparison with dogs fed the YCW food. Dogs fed the YCW diet tended to present lower IL-6 and TNF-α values than animals fed the control food (P<0.10). No differences were detected for the other cytokines evaluated. No differences between diets were verified for H_2_O_2_ or NO production for monocytes or neutrophils, as shown in (Table 9).

**Table 8:** Serum cytokines concentrations (pg/mL) of dogs fed diets with the addition of different yeast cell wall preparations (mean and standard error of the mean).

Item		Diets^1^		*p-value*
	CON	YCW	YCWs	
IL-6*	39.9^a^±8.8	22.7^ab^±9.5	21.0^b^±8.5	0.025
IL-10*	26.1±6.1	32.0±6.1	22.9±6.0	0.900
TNF-α*	37.1±10.0	24.1±10.3	33.8±10.1	0.081

^1^ CON = control, without yeast cell wall addition; YCW = 0.3% of a standard yeast cell wall extract; YCWs = 0.3% of a yeast cell wall extract with 20% soluble mannan.

* Values transformed to Log x + 1 or γ for statistical analysis.

^a, b^ = means in a row without a common superscript letter differ (P<0.05).

**Table 9:** Hydrogen peroxide (H_2_O_2_; μM of H_2_O_2_/2x10^5^ cells) and nitrogen oxide (NO; μM of NO/2x10^5^ cells) production in cell cultures of monocytes and neutrophils from the peripheral blood of dogs fed diets with the addition of different yeast cell wall preparations (mean and standard error of the mean).

Item	Diets^1^			*p-value*
	CON	YCW	YCWs	
H_2_O_2_ –Monocytes^2^				
Phorbol myristate acetate	2.0±0.2	2.0±0.2	2.1±0.2	0.921
Phorbol myristate acetate + Lipopolysaccharide	2.3±0.2	2.3±0.2	2.0±0.2	0.443
H_2_O2 –Neutrophils				
Only cells	0.5±0.1	0.5±0.1	0.8±0.1	0.118
Lipopolysaccharide	0.6±0.1	0.7±0.1	0.6±0.1	0.843
Phorbol myristate acetate	5.0±1.0	5.0±0.9	3.7±0.9	0.545
Phorbol myristate acetate + Lipopolysaccharide	3.2±0.6	4.4±0.6	3.0±0.5	0.161
NO–Monocytes				
Only cells	4.4±1.2	3.5±1.3	8.0±1.6	0.185
Lipopolysaccharide	5.8±1.7	6.0±1.7	4.3±1.7	0.806
NO–Neutrophils				
Only cells*	11.4±4.1	9.8±4.1	10.7±3.9	0.594
Lipopolysaccharide*	15.3±4.8	13.2±5.1	13.5±4.8	0.671

^1^ CON = control, without yeast cell wall addition; YCW = 0.3% of a standard yeast cell wall extract; YCWs = 0.3% of a yeast cell wall extract with 20% soluble mannan.

^2^ –For monocytes, the results of only cells and lipopolysaccharide were below the detectable limit of H_2_O_2_.

* Values transformed to Log x + 1 or γ for statistical analysis.

On day 30, the blood monocyte phagocytic index was 37% higher for dogs fed the YCW than the control diet (P<0.05), while dogs fed the YCWs food showed intermediate results. The neutrophil phagocytic activity of dogs did not differ among diets, having been elevated since the beginning of the study (Table 10).

**Table 10:** Monocyte and neutrophils phagocytic index (% of positive cells) from dogs fed diets with the addition of different yeast cell wall preparations (mean and standard error of the mean).

Item		Diets^1^			*p-value*
		CON	YCW	YCWs	
Neutrophils					
	Day 15*	89.1±2.4	89.9±2.4	92.1±2.4	0.807
	Day 30	84.9±1.6	88.9±1.6	87.7±1.6	0.189
Monocytes					
	Day 15*	47.6±2.0	48.2±1.8	49.8±1.8	0.579
	Day 30	54.8^b^±3.9	74.7^a^±3.8	64.4^ab^±3.8	0.010

^1^ CON = control, without yeast cell wall addition; YCW = 0.3% of a standard yeast cell wall extract; YCWs = 0.3% of a yeast cell wall extract with 20% soluble mannan.

* Values transformed to Log x + 1 for statistical analysis.

^a, b^ = means in a row without a common superscript letter differ (P<0.05).

## Discussion

Consistent with previous studies [40,41,42], the use of yeast cell wall was shown to be safe, as no changes in fecal quality or clinical condition of the animals was observed during the experimental period. The reduction of fat apparent digestibility after the consumption of the YCWs treatment can be attributed to the elevated solubility in water of yeast cell wall, perhaps behaving in the intestinal tract as a soluble and fermentable fiber that may interfere with fat absorption in dogs, as demonstrated by previous studies [43,44]. This effect on fat digestibility was not observed for the conventional YCW preparation, in agreement with previous publications on dogs [40,41]. The implications of the observed reduction in fat digestibility should be explored in future studies, including its use in low energy foods, but the magnitude of the fat digestibility reduction was low and its relevance to canine nutrition uncertain.

The experimental diets were formulated with sugarcane fiber, composed of approximately 45.8% cellulose, 28.1% hemicellulose and 9.3% lignina [45], an insoluble fiber source with very low fermentability [8,30] that was selected to not interfere with SCFA production. Under this condition, the intake of the more soluble yeast cell wall preparation, higher in soluble mannan oligosaccharides, changed the metabolism and fermentation products generated by the gut microbiota, as evidenced by higher fecal butyrate and putrescine and lower fecal lactate than in the other treatments. A higher production of SCFA, and especially of butyrate, is one of the outcomes expected from an effective prebiotic [13,46,47], suggesting an advantage for the YCWs. It is interesting that the traditional YCW, which is more insoluble, did not interfere with fermentation end-products formation; these data indicate that the solubility of the carbohydrate fractions of the yeast cell wall may be a key factor for product interaction with the gut microbiota.

In addition to its role as a source of energy for colonocytes, butyrate has been explored for its ability to directly affect cell growth and differentiation and to reduce cell inflammation [1,48,49]. In different cellular and animal models, butyrate reduced inflammation and improved the barrier function of the gut, reducing the production of proinflammatory cytokines [50,51,52]. Therefore, increased butyrate concentration is generally associated with improved health [53,54] and is one of the main objectives of prebiotic supplementations of diets.

Lactate is also produced as a result of carbohydrate fermentation by colon microbiota [55]; however, it does not exhibit a cumulative effect, as it is a substrate for several bacteria that utilize it, producing propionate and butyrate [56]. Thus, lactate concentrations may be interpreted considering the rates of production and consumption [56], which can explain the lower lactate and higher butyrate concentrations for dogs fed the YCWs diet. This altered the butyrate-to-lactate ratio, also exemplifying the impact of the YCWs on gut microbiota metabolism.

Amines are mainly formed through the decarboxylation of amino acids by the microorganisms of the gastrointestinal tract [57]. The fecal concentrations of amines observed in the present study are comparable to those previously reported in dogs [19,40,58,59], although the interpretation of amine concentrations in dog feces is difficult, due to the very limited information available regarding normal or desired levels [41]. In the present study, as the protein source (a possible source of amines) in diets was the same, the increased putrescine concentration (and the tendency of increased spermidine) may be explained by higher intestinal formation after the intake of the YCWs. Putrescine is produced by the decarboxylation of ornithine and arginine and, in turn, is progressively metabolized to spermidine, justifying the concomitant increase of both amines [60]. A previous study in our laboratory did not find an effect of yeast cell wall on fecal amine concentrations [41], reinforcing the lack of effect of the YCW diet in the present study, and suggesting that the soluble mannan oligosaccharides fraction of the YCWs in fact altered the fermentation profile of the gut microbiota.

Several favorable and harmful physiological processes involve the action of amines, especially the polyamines [61]. They are present in all living cells and are necessary for the normal development and repair of intestinal mucosa cells [62,63]. However, their activity has also been associated with the incidence of colorectal cancers [64], and high concentrations are related to inflammation, oxidative stress and genotoxicity [65]. Therefore, a significant reduction of polyamine concentrations in the intestinal lumen is not interesting, since the polyamine depletion (intracellular) directly affects the apoptosis of epithelial cells [40]; however, high amounts may also be undesirable. In a study with dogs in different age groups, higher putrescine, cadaverine, and spermine were observed in feces of older dogs compared to adult dogs, and higher spermidine was found in feces of dogs fed a diet based on soybean meal, which was linked by the authors to a higher IgA content in feces and better intestinal health [3].

IgA was evaluated in the present study, as it is an important marker of the mucosal immunity status [42,66], representing an essential factor in the protection against infectious agents, allergens and foreign proteins [3,67,68,69]. The main function of secretory IgA is to prevent bacteria and viruses from attaching and invading enterocytes [70,71]. The evaluation of this immunoglobulin is also of interest to clinicians to assess specific responses to antigens or in the diagnosis of IgA deficiency [66]. Studies with newborn animals have shown an effect of YCW on IgA secretion [72], differing from the present results. Perhaps the use of animals with mature immunity and the lack of immunological challenge in the present study may have interfered with the evaluation of the possible effect of the yeast cell wall preparations on the secretion of IgA, as also observed when the prebiotic resistant starch was evaluated in healthy adult dogs [73].

Cytokines was only evaluated after 30 days of diet intake, and due this was not possible to evaluate the kinetic of these compounds. The reduced IL-6 in serum of dogs fed the YCWs diet may also result, at least partially, from the higher butyrate formation in the intestine. Butyrate appears to be more potent than acetate or propionate in inducing immunomodulatory effects, as it affects the activity of histone deacetylases, which are responsible for decreasing the secretion of IL-12 and IL-6 cytokines by dendritic cells and allow dendritic cells to enhance mucosal regulatory T-cells [74]. However, a tendency for lower IL-6 and TNF-α in serum was also verified for dogs fed the YCW diet, which did not alter SCFA fecal concentration. These data corroborate findings of other researchers [75], which evaluated the action of a yeast cell wall fraction called "mannoprotein", added to a liquid diet for rats with Salmonella infection. The authors also found lower expression of TNF-α and IL-6 mRNA in the jejunum, ileum and colon tissues in the treatment groups and concluded that yeast cell wall derivates may lower the inflammatory response, protecting the intestinal tissue. Therefore, one may speculate that YCW may have a direct action on intestinal cells, reducing proinflammatory cytokines, in a mechanism independent of SCFA formation.

The increase in peripheral monocyte phagocytic activity in dogs fed the YCW diet was relevant, as this phenomenon is an important criterion for evaluating innate immunity [76,77]. Phagocytic cells act as the first line of defense against microorganisms [78], with monocytes being the key mediators of the early inflammatory response to infection. Considering the lack of changes in fermentation products in feces, it is possible to attribute this effect to a direct interaction of the mannan oligosaccharide or the b-1.3/1.6 glucan fractions of the YCW with the dendritic cells of the intestinal mucosa [79,80] which could demonstrate the ability of the YCW to modulate the immune system directly [49]. The ability of b-glucans to increase monocyte and neutrophil phagocytic percentages is well demonstrated for several species, including dogs [77,81,82]. However, a direct interaction of the mannan oligosaccharide fraction and the immune system has also been described [83,84] and cannot be excluded.

Peripheral blood mononuclear and polymorphonuclear (neutrophils) cells are traditionally used to evaluate *in vitro* responses of blood-derived immune cells to various antigens [85]. Although in the present study cells were stimulated by lipopolysaccharide and phorbol myristate acetate and substantially increased H_2_O_2_ and NO production, no diet effect was verified. Several studies regarding dietary intervention also found no effect on H_2_O_2_ or NO production [65,85]. The procedure is laborious, expensive and requires large volumes of blood to obtain the appropriate number of cells. In addition, cell sorting can stimulate cells and lead to loss of specific populations, leading to results that may not reflect the condition *in vivo* [85].

Some limitations of the present study may need to be considered. Only healthy dogs were used, and the period of dietary intake was not previously studied to determine if it was sufficient to express the complete effects of both yeast cell wall preparations. Consequently, possible differences between groups in the immunological system and microbiota metabolism could not be observed, and the long-term effects of the products are not known.

## Conclusion

Under the conditions of the present research, positive immunomodulatory effects were verified for both yeast cell wall preparations. The addition of YCWs to an extruded diet changed intestinal microbiota metabolism, as verified by increased butyrate and putrescine and reduced lactate. YCWs in the diet also reduced inflammatory markers, which was verified by a reduction of serum IL-6 in dogs. The conventional YCW also tended to reduce IL-6 and TNF-α, and stimulated innate immunity, verified by an increase in peripheral monocyte phagocytic activity.

## Supporting information

S1 Table Full data set. CON = control, without yeast cell wall addition. YCW = 0.3% of a standard yeast cell wall extract.YCWs = 0.3% of a yeast cell wall extract with 20% soluble mannan oligosaccharides.VFA = volatile fatty acids. SCFA = short-chain fatty acids. BCFA = branched-chain fatty acids.
(XLSX)
